# Incorporating acoustic objectives into Forest Management Planning when sensitive bird species are relevant

**DOI:** 10.7717/peerj.6922

**Published:** 2019-05-16

**Authors:** Carlos Iglesias-Merchan, Esther Ortiz-Urbina, Marta Ezquerro, Luis Diaz-Balteiro

**Affiliations:** 1 Department of Forest and Environmental Engineering and Management, Universidad Politécnica de Madrid, Madrid, Spain; 2 CENERIC Research Centre, Tres Cantos, Spain

**Keywords:** Biodiversity, Forest management, Forest logging, Noise mapping, Cinereous vulture, Environmental noise, Noise modeling, Natural resources management

## Abstract

**Background:**

The potentially negative effects of timber harvesting on biodiversity and habitat conservation leads to the consideration of a wide range of restrictions to forest logging in natural areas. In particular, high noise levels produced by forest machinery present a challenge to developing sustainable forest management plans. The Cinereous vulture (*Aegypius monachus*), the largest bird of prey whose nests are located in mature trees, is considered to be appropriate as an indicator species for environment-friendly forest planning. In this work, we evaluated spatially differences in sound propagation between stands. We hypothesized that differences due to the influence of orography in mountainous forests would allow the relaxation of spatial and temporary restrictions to timber logging, without causing any great disturbance at nesting sites of sensitive species.

**Methods:**

Our study was conducted in a Scots pine (*Pinus sylvestris*) forest of Spain, where an important colony of the Western European population of Cinereous vulture is located. We built 62 noise maps to characterize noise pollution due to tree logging at planning level. We modeled two different scenarios, in order to characterize; (i) the effect of a chainsaw operator during a complete cycle for felling a tree (Scenario 1), and (ii) the effect of the peak level produced by the breaking noise emitted by the trunk of the tree and its impact on the ground (Scenario 2). A strategy of three logical steps was designed; (i) landscape-scale analysis of noise propagation in stands, (ii) hierarchical cluster analysis of stands, (iii) assessment of the potentially significant influence of noise management in timber harvesting.

**Results:**

The minimum distance (*DIST*) from chainsaw operator sites to the 40 dB(A) contour lines was the only variable that had a significant influence on the clustering results. On the other hand, mean values of a newly proposed metric called average radius or radial distance (ARD) oscillated between 174 m in cluster #1 (Scenario 1) and 407 m in cluster #2 (Scenario 2).

**Discussion:**

Our results highlight the convenience of considering noise modeling tools at a forest planning level in order to address the compatibility of forest logging and the necessity of protecting nature. We found that spatial propagation of noise made by chainsaws at felling sites does not differ between stands even in a mountainous terrain, contrary to what we initially hypothesized. However, temporary logging restrictions could be excluded in about 36% of the current conditioned management areas according to *ARD* results in Scenario 2 (400 m). This proposal could be based on a sound pressure level (in decibels) criteria instead of conventional buffer protection distance criteria. In addition, it is suggested that the current size of restricted management areas could be generally extended from a 100 m radius to a 200 m one around the Cinereous vulture nest sites.

## Introduction

Human disturbance of wildlife is a growing concern in biodiversity conservation as recreational and industrial uses of natural areas are continuously expanding (Gill, Sutherland & Watkinson, 1996). In many cases, changes in habitat quality restrict access to resources such as food supplies or nesting sites (Gill, 2007). Commercial logging alters habitat structure and it can directly influence the occurrence of animal communities, although post-treatment stands are not necessarily uninhabitable (Guénette & Villard, 2005; McDermott & Wood, 2010). Indeed, logged forests may also have an essential role in conservation (Johns, 1985), and it is worth mentioning that species composition shifts naturally during a stand development (Reich et al., 2001). However, biodiversity conservation is a significant concern in sustainable forest management and the best management practices for timber harvesting (Ezquerro, Pardos & Diaz-Balteiro, 2019). Silvicultural treatments vary in intensity and a fundamental component of timber production is to leave trees of importance to flora and fauna at logging (Gustafsson, Kouki & Sverdrup-Thygeson, 2010). Nevertheless, it can significantly reduce the abundance or productivity of certain species (Guénette & Villard, 2005).

Relict wooded areas are considered as being the last haven for many endangered species (Donázar et al., 2002), and the management of a site is often targeted toward particular ones (Gill, 2007). This is an increasingly required practice that may vary from leaving single trees to retaining patches and buffer zones when cutting. In this sense, retention patches are assumed to maintain species richness better than solitary trees (Gustafsson, Kouki & Sverdrup-Thygeson, 2010). However, land management guidelines often lead to inappropriate generalizations of conservation paradigms due to the scarcity of evidence on this view (Schmiegelow & Mönkkönen, 2002). Mostly, the responses from bird communities have been used for assessing interactions between wildlife and timber harvesting because of their sensitivity to changes in forest structure (Plumptre & Johns, 2001). Among birds, some taxa such as raptors could be appropriate as indicator species for monitoring changes on large spatio-temporal scales. In short, understanding the relationship between forest management and bird populations requires including those indicator species that are sensitive to management issues (Carrete et al., 2009; Morris et al., 2013).

In some regions, many forests have been managed to protect habitats for endangered and threatened species, such as Spotted owl (*Strix occidentalis*) in the United States. The latter has become an icon of conservation conflict for more than three decades, and logging restrictions due to owl protection have had serious socio-economic ramifications (Redpath et al., 2013). In Europe, silvicultural practices have been pointed to as being a reason for understanding differences in nest-site selection between colonies of Cinereous vulture (*Aegypius monachus*) (Fargallo, Blanco & Soto-Largo, 1998). The latter is the largest bird of prey (Falconiformes) in the world and its breeding distribution in Europe is limited to some parts of Spain, Portugal (inland), France (as reintroduced between 1992 and 2004), and southeastern European regions (Greece and Turkey). Furthermore, Cinereous vulture nests in mature trees and conservation and management guidelines for this species usually include protecting its suitable habitat from disturbance and logging (Poirazidis et al., 2004).

Accordingly, buffer zones around nest sites and retention in timber volume are already frequently applied as a measure to preserve Cinereous vulture populations in Spain, even without defining then as such in forest management plans (Ezquerro, Pardos & Diaz-Balteiro, 2019). Nevertheless, researchers have not individually assessed the real influence between the several causes of potential disturbance due to logging (Plumptre & Johns, 2001). Today, there is a need for research to identify threshold ranges in habitat alteration that can be tolerated by the most sensitive species (Guénette & Villard, 2005), as well as the way in which disturbance due to human activities may interact synergistically or additively with habitat structure alteration (Michalski & Peres, 2017). Therefore, managing forests to fulfill both production and conservation goals is a growing challenge around the world (Donázar et al., 2002). In particular, it challenges planners to find thresholds in relation to forest harvesting restriction limits, taking into account sound propagation and its potential of disturbance. In some managed forests in Spain, forestry operations are forbidden on a 500 m radius circle (78.54 ha), within the so-called conditional management area (CMA), in order to prevent disturbance due to forest machinery during the breeding season. So, forest workers such as chainsaw operators can only operate for approximately 3 months per year. In addition, a stricter buffer protection radius of 100 m (called restricted management area, RMA) is left and not usually cut around each Cinereous vulture’s nest (Ezquerro, Pardos & Diaz-Balteiro, 2019).

Forest operations involve the use of machines producing noise, and noise disturbance from machinery may alter animal behavior and their spatial and temporal dynamics (Turner, Fischer & Tzanopoulos, 2018). However, the forest research community’s attention has been mainly focused on the potential health problems of workers due to their exposure to the high noise levels of many forestry machines (Neitzel & Yost, 2002; Kováč et al., 2018). Consequently, efforts to reduce occupational exposure to noise and vibrations have increased substantially during decades (Rottensteiner et al., 2013). Thus, previous research has reported that the highest values of noise pollution are caused by chainsaws at felling sites above all other forest machines (e.g., forwarders, loaders, harvesters, processors and skidders) during normal operational activities (Sowa & Leszczyński, 2007; Tunay & Melemez, 2008; Fonseca et al., 2015; Proto et al., 2016).

In another direction, exposure of wildlife to noise pollution has been subject of research on transportation networks, which are considered to be the most pervasive source of anthropogenic noise (Barber, Crooks & Fristrup, 2010). In some cases, researchers estimate disturbance distances at several hundreds of meters from noise sources. However, it was pointed out that very long disturbance distances might be unrealistic and they rarely exceed 1,000 m (Reijnen, Foppen & Veenbaas, 1997). Besides, terrain topography may not be flat, leading to shadow zones due to the presence of visual and acoustic barriers and obstacles, which also reduce reaction probability (Embleton, 1996; González et al., 2006). However, there is a large variety of parameters relevant to sound propagation (e.g., ground absorption, type of noise source, duration, etc.), which we cannot obviate (Murphy & King, 2010). Thus, the Euclidean distance to the noise source may not be an appropriate index for assessing wildlife disturbance due to human-made noise (Shannon et al., 2016; Iglesias-Merchan et al., 2018).

In this sense, Iglesias-Merchan, Diaz-Balteiro & De la Puente (2016), found the total absence of any Cinereous vultures nesting within the *L*_eq_ level of 40 dB(A) in the surroundings of a mountainous road in Lozoya Valley (Spain). Their results referred to decibel contour lines instead of distances in meters because there was not a single Euclidean distance related to the same sound pressure level (SPL). Indeed, the 40 dB(A) contour line ranged between a minimum value of 75 m and a maximum value of 500 m from road margins where no Cinereous vulture nests were found, and suggested a potential breeding exclusion area in terms of SPL. In mountainous terrains, SPL contour lines may be plotted as highly convoluted contour lines in noise maps, unlike the more typical parallel contour lines from the noise source margins, that are better suited to those in flat terrains with an absence of obstacles to sound propagation (Steele, 2001; King & Rice, 2009). Furthermore, Shannon et al. (2016) found, in a review of two decades of research studies, that the most frequent terrestrial wildlife responses begin at a noise level of approximately 40 dB(A). However, long-term studies will be necessary to assess any chronic effects (Yorzinski & Hermann, 2016).

Noise maps are an important tool for providing relevant information on environmental impacts and enabling the visualization of noise propagation through the landscape (Iglesias-Merchan & Diaz-Balteiro, 2013; Bastián-Monarca, Suárez & Arenas, 2016). Also, noise mapping, supplemented with spatial pattern analysis, is considered to be a helpful way to predict and assess present and future areas of conflict with human activities at large scales (Iglesias-Merchan, Diaz-Balteiro & Soliño, 2015). However, there is a lack of use of environmental noise prediction tools to explore noise propagation in forest management in spite of it being the main reason for restricting timber harvesting in forest areas with presence of endangered wildlife species. Therefore, the main objective of this research was to analyze sound propagation through different stands and to compare spatial patterns of chainsaw noise propagation with the current restrictions to forest logging in order to prevent noise disturbance on nesting sites of the Cinereous vulture. We hypothesized that it would be possible to classify stands into different groups, in which different buffer protection distances (BPD) could be defined around the nests. This is because of the different orography of stands and their response to sound propagation, which, in turn, could relax spatial and temporal restrictions to timber logging of forest planners without causing a greater disturbance to nests. Consequently, a second objective of this study is to predict an average BPD on each stand according to their orography conditions and the previous results for sound propagation. Therefore, in this study we have built 62 noise maps to model and assess noise propagation from chainsaw operators during tree-felling in a managed forest area in Spain, where the conservation of protected sensitive bird species is a key objective of forest management plans.

## Material and Methods

### Study area

**Figure 1:**
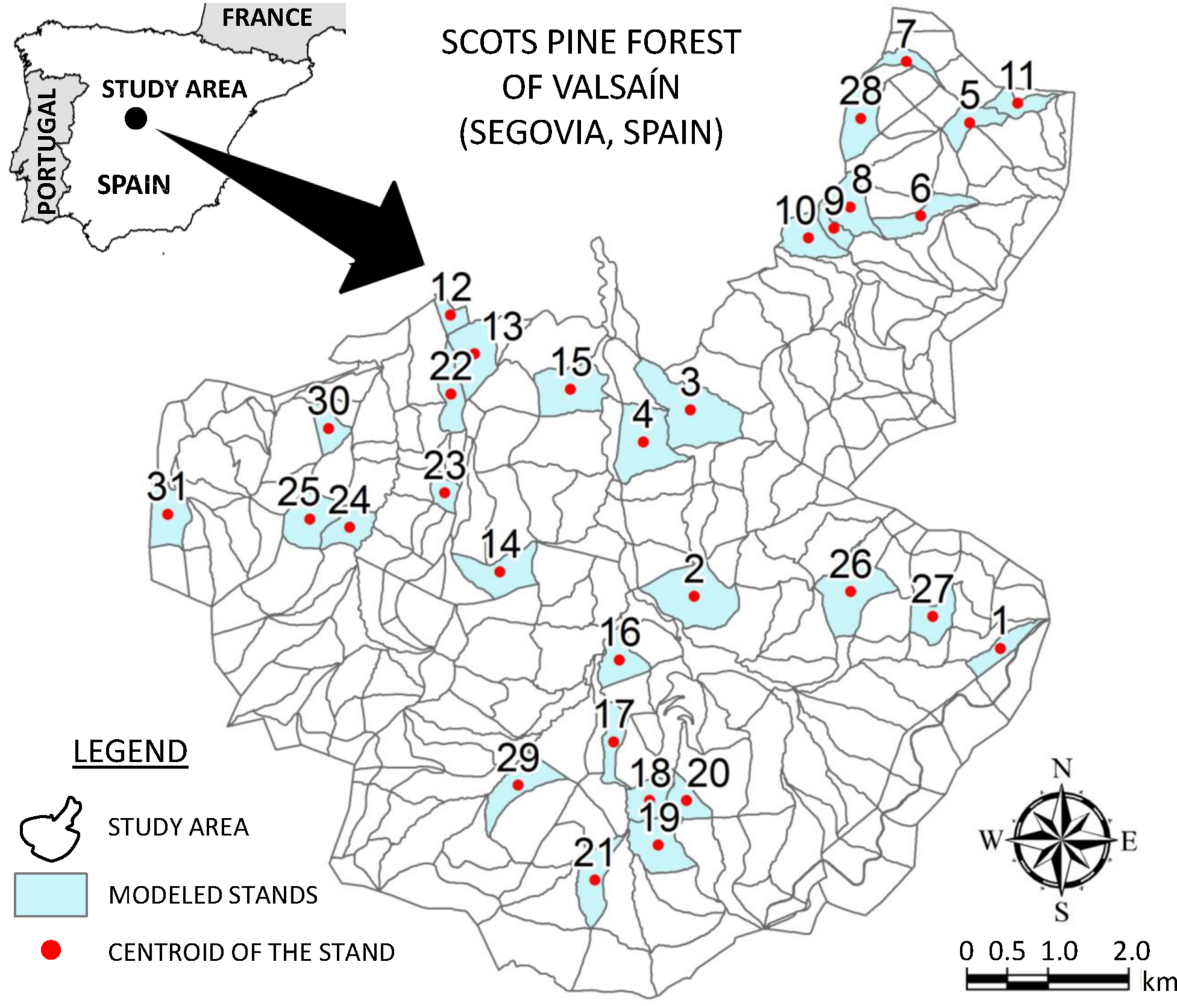
Study area location.

The present study was conducted in the forest of Valsaín (province of Segovia), under the jurisdiction of Sierra de Guadarrama National Park managers in the Central Mountains of Spain (Fig. 1). The main tree species is Scots pine (*Pinus sylvestris*), that covers about 7,500 ha of this area. The forest belongs to the state and its first forest management plan dates from 1889. Regarding its spatial organization, the forest is formed by 288 stands and the current forest management plan (2010–2020) pays close attention to aspects like biodiversity conservation (Diaz-Balteiro et al., 2017). Indeed, there is an important Cinereous vulture breeding colony in the forest of Valsaín, where approximately 131 nests (official data) have been inventoried. Cinereous vulture is a species classified as being highly threatened at the European level, considered near-threatened by the International Union for Conservation of Nature, IUCN (2006 IUCN Red List of Threatened Species), and listed in Appendix I of the CITES (Convention on International Trade of Endangered Species of Wild Fauna and Flora) (Poulakakis et al., 2008; Mihoub et al., 2014).

Traditionally, illegal poisoning and disturbances in breeding areas have been highlighted as being causes of the species decline, but long-term research on the impacts of timber felling on birds of prey is limited (Donázar et al., 2002). Anyway, local populations are considered likely to be sensitive to disturbance, among other factors, and this species needs large areas of an appropriate habitat for its well-being (Carrete & Donázar, 2005; Morán-López et al., 2006). Therefore, the prohibition of mature tree-cutting is a very common management measure within strict protection zones, as well as implementing a rigid control of non-paved accesses to prevent disturbance (Poirazidis et al., 2004). In the forest of Valsaín, RMA zones are defined by a 100 m radius buffer protection around each nesting site, which is left and not usually cut. In addition, logging and other disturbing activities are forbidden in the CMA zones (on a 500 m radius circle) during the breeding period (9 months, from January to September). Consequently, forestry operations are restricted to a 3 month period (fall) within CMA zones and this triggers serious socio-economic outcomes (Ezquerro, Pardos & Diaz-Balteiro, 2019). The latter illustrates one of the most difficult challenges for conservation biology; to reconcile the exploitation of natural resources with the rising need to protect nature (Rosin et al., 2016). Indeed, the confluence of constraints such as low-intensity systems, low incomes and the physical disadvantage of mountain areas, frequently lead to land abandonment that may cause a corresponding loss in natural capital that needs to be addressed (MacDonald et al., 2000).

In particular, the study area is defined by 31 stands of the forest of Valsaín (Fig. 1), which correspond to those in which cuts have been scheduled for the first 10-year period in the strategic model planned by Ezquerro, Pardos & Diaz-Balteiro (2019). Current forest planning is based on a uniform shelterwood system with a rotation of 120 years and, since the 1980s, an extended regeneration period of 40 years (Pardos et al., 2016).

### Noise modeling

#### Sampling method

There was no previously consolidated sampling methodology to be followed in the conducting of studies as we had intended. So, taking into account the landscape scale, initially, we decided to select a characterizing single point to represent each stand and its centroid point was chosen for this purpose. In addition, it was also important to consider that forest stands have largely been managed as a relatively homogeneous contiguous area (Donázar et al., 2002; Boyden et al., 2012). These spatially functional management units usually have a common set of characteristics (height, density, age, structure, species composition), not only because of their silviculture but also the physical site variables such as elevation, topographic exposure and slope (Urban et al., 2000).

However, realizing that our study might not adequately reflect the intra-stand variability, we conducted a previous comparative analysis of topographical variables at the stand level. We used ArcGIS 10.6.1 (Esri, Redlands, CA, USA) to calculate data for the next three orographic variables from the digital elevation model: (1) slope of the ground (*SLOPE*, in percentage), because the terrain’s orography influences forest composition and noise propagation (Urban et al., 2000; Guarnaccia, Mastorakis & Quartieri, 2011); (2) ground elevation (*ELEV*, in meters) that also influences forest composition; (3) the slope aspect (*ASPECT*, in degrees), which is the compass direction that a slope faces, because forest structure is frequently different, depending on the topographic factors (Lindenmayer et al., 1999). The orographic data were calculated, firstly, at each stand’s centroid point and, secondly, the average values of *ELEV*, *SLOPE* and *ASPECT* within each whole stand’s surface. Average values in each stand were calculated from a grid of points (10 × 10 m resolution) that covered the study area.

**Figure 2:**
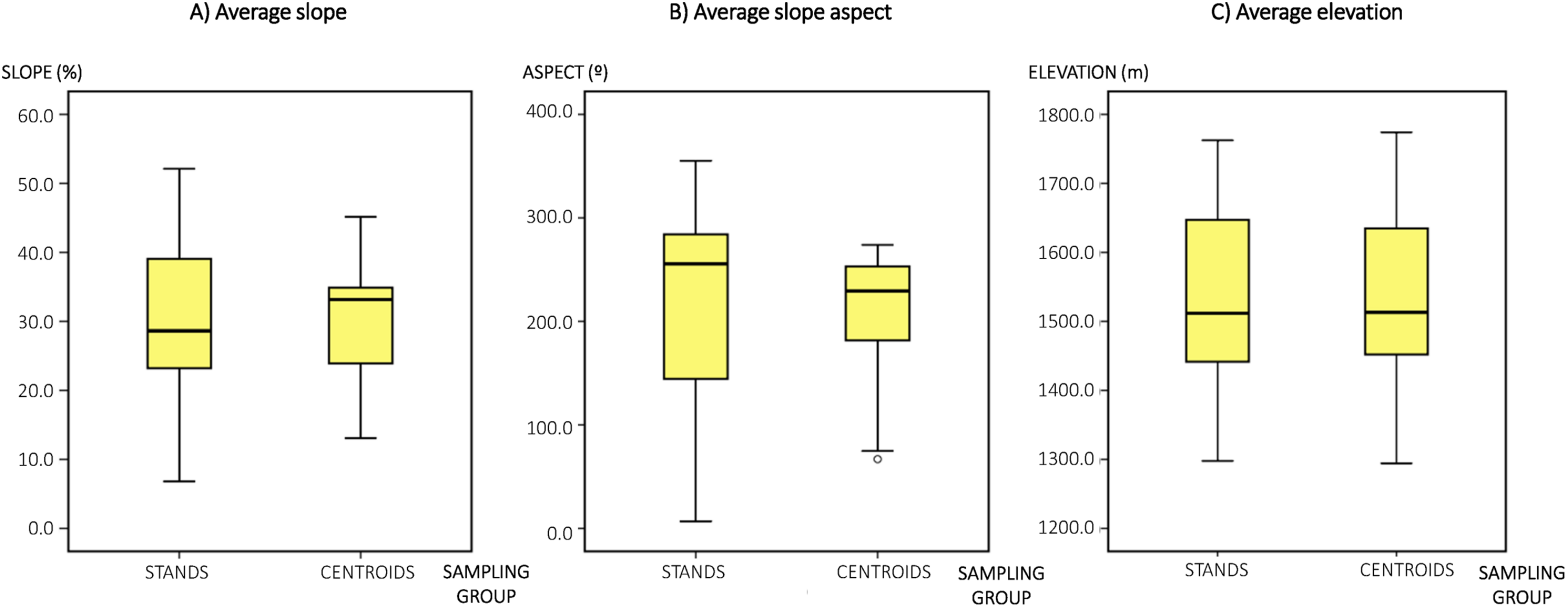
Box-plots diagrams. (A) Average slope (percentage) within each stand surface area and at each stand’s centroid point. (B) Average slope aspect (degrees) within each stand surface area and at each stand’s centroid point. (C) Average elevation (meters) within each stand surface area and at each stand’s centroid point.

A preliminary Kolmogorov–Smirnov test revealed that *ASPECT* and *SLOPE* data were not normally distributed. Therefore, we used the nonparametric Mann–Whitney *U* test to compare homogeneity of orographic data between centroid points of stands and the rest of each stand. We found that there were no statistically significantly differences in slope aspect and elevation between centroid points and average values within stands (Mann–Whitney *U* test: *U* = 390, *p* = 0.203 and *U* = 467, *p* = 0.849, respectively). On the other hand, the *ELEV* dataset fulfilled the assumption of normal distribution and equal variances (Levene test), so that a paired sample *t*-test was used and there was no statistically significant difference in *ELEV* values between the two groups (*t* (60) = −0.051, *p* = 0.960). Therefore, given the intra-stand topographical homogeneity (Fig. 2), we decided to maintain a sampling scheme for building noise models based on the stands’ centroid points as being sufficiently representative of every stand’s hypothetical response to sound propagation. Namely, especially taking into account that we were seeking and assessing the potential use of noise modeling at a reasonable spatial scale for the decision-making process in forest planning, instead of a further detailed design of operations at the stand scale.

#### Noise modeling: general settings

Spatial noise models were set up in order to investigate noise propagation due to chainsaws during timber logging within the study area. Noise modeling allows the assessment of environmental noise, in terms of SPL, over a limited area defined by a calculation grid. Noise modeling was based on the European Directive on environmental noise, END (European Council (EC), 2002). Despite the END being intended for human receivers, it has been successfully adapted to assess the potential impact of anthropogenic noise sources in a variety of wildlife species (Iglesias-Merchan, Diaz-Balteiro & De la Puente, 2016; Iglesias-Merchan et al., 2018; Martínez-Marivela et al., 2018). END (European Council (EC), 2002) establishes that noise maps for a local or national application must be made for an assessment height of four m and the five dB(A) ranges of *L*_den_ and *L*_night_ indicators as defined in ISO 9613-2 (1996). Nevertheless, a feasible strategic noise mapping adaptation would be to adopt the equivalent continuous SPL (*L*_eq_) as a reliable noise indicator (Pater, Grubb & Delaney, 2009). In this case, we prefer to use the *L*_eq_ index because it does not include human-perceived subjective penalties, such as, for instance the 24-h noise indicator (*L*_den_) recommended by END (Paunović, Jakovljević & Belojević, 2009). Besides, the *L*_eq_ level (in decibel, dB) can be calculated to assess disturbance due to anthropogenic noise during different periods of time instead of 24 h (Martínez-Marivela et al., 2018). Furthermore, *L*_eq_ measurements or computation methods can be adjusted depending on the periods of human activity or the wildlife activities to be monitored (Shannon et al., 2016) and it was our desire to model a tree-cutting period of time.

We considered the worst-case parameters regarding weather conditions in calculation settings, taking into account the ISO 9613-1 (1993) standard. So, we used the values of average monthly maximum temperature and average relative humidity in October (11 °C and 79%, respectively), based on meteorological data from Navacerrada Pass weather station (State Meteorological Agency, AEMET). In addition, ground absorption was considered by default as porous soil (coefficient *G* = 1), because it corresponded to normal un-compacted ground types (forest floors, pasture field) in accordance with the acoustic characterization of grounds included in Kephalopoulos, Paviotti & Anfosso-Lédée (2012). Finally, foliage areas were also considered in model calculations and we took into account average tree height at stand level (official data from the current forest management plan).

Sound pressure levels were calculated over a grid of 10.00 × 10.00 m located in a 500 m radius buffer around each stand centroid and at a height of 4.00 m above ground level in accordance with Iglesias-Merchan, Diaz-Balteiro & De la Puente (2016) and the END (European Council (EC), 2002). A grid consists of a set of receiver points where SPL is calculated, and it is used as the basis for SPL contours (isophones) when plotting noise maps. The noise maps were produced by the *Computer-Aided Noise Abatement* software (CadnaA Version 2018 MR 1, 32-bit) software, taking into consideration the ISO 9613-2 (1996) interim computation method, a very common industrial noise prediction method and also the one advocated by END (European Council (EC), 2002) whose recommendations were followed. Many industrial noise sources exhibit substantial directivity; however, the majority of methods for industrial noise modeling do not specify much detail on the input and application of directivity (Datakustik, 2017), and no directivity corrections were adopted (uniform radiation). In the case of point sources, CadnaA considers that the directivity vector points to the normal direction of the nearest reflecting façade (in this case the ground should be seen as a reflection surface). Lastly, the information required for the noise models included the digital elevation model, which was based on the official 1:25,000 scale topographic digital maps (10 m contour lines) from the National Geographic Institute (Instituto Geográfico Nacional).

#### Noise source characterization

Continuous or fluctuating and impulse noise which take place simultaneously are the most common types of noise caused by the use of industrial devices (Żera, 2001). Impulse sound refers to a high intensity signal of a short duration (sometimes less than a second) and most countries limit impulse noise exposure in their occupational legislation because of their potentially negative effects on workers’ health (Arenas & Suter, 2014). However, there are no references or standards for the measurement and assessment of impulse noise in relation to its conceivably negative effects on wildlife in natural areas. Among the variety of sound level descriptors, one important parameter describing the level of impulse noise is the C-weighted peak SPL (LCpeak) or linear peak (unweighted) SPL (Lpeak) (Żera, 2001).

**Figure 3:**
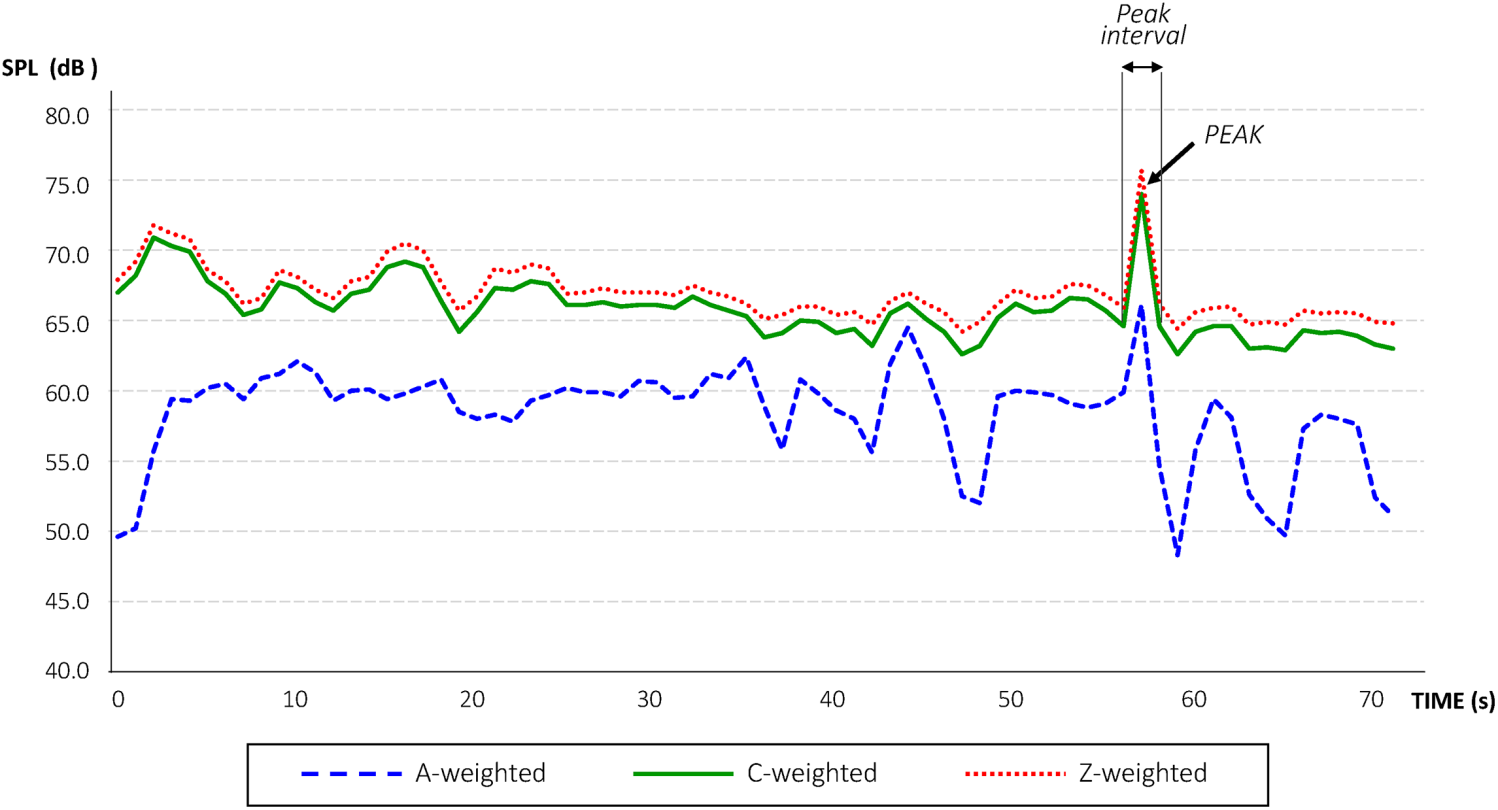
Level-time curve of the sound pressure level produced by a tree-felling with a chainsaw.

To characterize the sound power level of chainsaws we conducted our own field measurements, which were taken in the fall of 2017 (field study approval number “Expte: PNSNG_SG_2017_0180,” authorized by the Service of the Environment, Regional Government Delegation in Segovia, The Government of Castilla y León). Noise field measurements were conducted successfully from 30 to 90 m distance from chainsaw operators for safety reasons. A professional sound level meter CESVA SC 420, which meets the specifications of IEC 61672 international standard for class1, and the American standards ANSI S1.4 and ANSI S1.43 as class 1, was employed. The total equivalent noise level in second intervals was recorded, as well as equivalent noise level by 1/1 octaves of frequency spectrum. The frequency spectrum of field noise measurements ranged from 16 to 16,000 Hz. The sound level meter measured all the functions simultaneously with all frequency weightings (A, C and Z), and calculated statistical data, such as maximum and minimum values and percentiles. It also measured certain sound events every 125 ms to analyze sounds that may be particularly short in duration. A windscreen was used and the microphone was placed at approximately 1.50 m above the ground. In addition, the meter was calibrated before and after every recording period, and records were only accepted if the calibration reading deviations were lower than ±0.5 dB. Temperature, humidity and wind speed during the field measurements were also monitored to comply with the device’s weather-threshold specifications or methodological requirements. Furthermore, a digital voice recorder was simultaneously used for sound capture and noise-source deskwork verification. According to our field observations, the noise source was placed at an average height of 0.30 m above the ground level in noise modeling. Consequently, based on our field measurements, we were able to obtain the spectral content of the tree-felling signal (Fig. 3) and to measure both indices *LCpeak* and *LZpeak* during field work.

In this way, it was possible to estimate the sound power level characteristic of a complete tree-felling cycle as well as the sound power level of the peak signal (Table 1). In this step, there is a deficit in the literature of studies on species-specific weighting and thresholds of audibility based on audiograms and known sound stimulus. However, a case study can be found that is based on the response of the Spotted owl to the sound spectrum of a helicopter, which shows that A-weighting matches the owl-weighted function better than the unweighted curve (Pater, Grubb & Delaney, 2009). In the absence of any evidence on this matter, we decided to conduct our research in A-weighted decibels, also because it is possible to find noise metrics in dB(A) with respect to anthropogenic disturbance and wildlife behavioral and physiological responses (Iglesias-Merchan et al., 2018). On the other hand, the use of common tools and frequency weighting may make research gain more options in terms of their future applicability by stakeholders in a variety of fields. Thus, we determined that sound power level (A-weighted) linked to the use of chainsaws was approximately the same (115.7 dB(A)) as maximum levels guaranteed by the most popular chainsaw manufactures (referring to the ISO 9207 standard) in the case of petrol chainsaws for forestry within the power output interval of between 4.4 and 6.4 kW. However, the sound power level associated with the peak sound, caused by the succession of the final breaking noise emitted by the trunk of the tree and the impact sound of the same tree trunk hitting the ground was much higher (125.4 dB(A)) than the maximum emission level of this type of forestry machinery. Thus, we considered that both sounds (the complete cycle and the particular peak noise) should be taken into account in our research.

**Table 1:** Frequency spectra (in octave bands) of the modeled noise sources.

Frequency band (Hz)	31.5	63	125	250	500	1,000	2,000	4,000	8,000	Total dB(A)	Total dB
Tree cutting	111.9	109.9	109.7	115.2	115.3	107.1	106.1	105.1	103.6	115.7	120.7
Trunk breaking & hitting ground	121.6	113.7	120.9	121.0	127.0	117.9	110.2	108.0	106.3	125.4	130.0

**Note:**

Tree cutting, shows the sound power level and frequency spectra during a complete chainsaw operation cycle for felling a tree; Trunk breaking & hitting ground, show the sound power level and frequency spectra of the peak noise event due to the tree trunk breaking and its impact on the ground; Total dB(A), the A-weighted total sound power level; Total dB, the linear (unweighted) total sound power level.

In total, we built 62 noise maps corresponding to two different scenarios in the above-mentioned 31 stands, called Scenario 1 and Scenario 2. In Scenario 1, we built 31 noise maps on which we plotted the effect of sound propagation made by a chainsaw operator when felling a tree. This means that, in Scenario 1, we considered the complete cycle of the cutting operation, that frequently lasts 1 or 2 min. These noise maps showed *LAeq*_*t*_ levels, which is the equivalent continuous sound level during the tree-felling period of time (indicated by the sub-index “*t*”) by a chainsaw operator (in A-weighted decibels) at a buffer radius of 500 m around each centroid point. In one sense, this is also a particular worst-case to assess noise pollution from chainsaws against to model a time interval of an hour of work or the regular working hours. The last case implies a reduction in tree-felling efficiency of approximately 25% of the time interval in a Scots pine forest (Ambrosio, Tolosana & Vignote, 2001), because of the breaks into the working day to allow workers to eat and to rest, which would imply a correction toward lower sound power levels.

In Scenario 2, we built 31 more noise maps on which we plotted the *LApeak* level, which is the peak SPL (125.4 dB(A)) due to the loud typical breaking noise of wood emitted by the tree trunk just before the next impact sound of the same tree hitting the ground. This event lasts approximately 2 or 3 s in all. All the relevant parameters selected in the calculation settings remained by default as they were stated in Scenario 1. We even decided to keep the noise source at an average height of 0.30 m above the ground level. This is because the event is defined by a combination of two sounds; the first one is caused when the trunk of the tree cracks (approximately at the height of the wedge-shaped first cut) and the second, obviously, when the trunk hits ground level.

### Spatial pattern analysis

We exported noise maps to a geographic information system (GIS), allowing us to conduct a geostatistical analysis of SPL results. Landscape ecology enhances the measurement of landscape structure in meaningful ways that, making it possible to understand ecological processes (Turner, 1989). Landscape structure refers to the composition and configuration of landscape patterns and numerous indices have been developed to quantify landscape structure changes (Paudel & Yuan, 2012). Indeed, the amount of indices is often overwhelming even for landscape ecologists (Li & Wu, 2004). Thus, we considered it to be of great assistance to use a limited number of indices for assessing the level of hypothesized heterogeneity in the structure and composition of the resulting patches of equal SPL in noise maps. SPLs are frequently represented by categories defined by ranges of five dB in noise mapping. Therefore, area metrics can be known immediately but we considered it to be of interest to compare the number of the resulting polygons and their complexity in terms of shape and fragmentation for exploring the geometry of the propagation of tree-felling noise through the forest acoustic environment. Therefore, five of the most common metrics (as defined by McGarigal & Marks, 1995) were employed (see definitions in Table S1), grouped into the following useful categories for noise polygon pattern analysis (Table 2): number of patches (*NumP*), mean patch size (*MPS*), edge density (*ED*), area-weighted mean shape index (*AWMSI*) and mean patch fractal dimension (*MPFD*). Landscape indices or spatial statistics were calculated using Patch Analyst 4.2.13 for ArcGIS 10.6.1 (Esri, Redlands, CA, USA) in order to quantify changes in sound propagation patterns. Patch analyst is a program which facilitates spatial analysis and the modeling of attributes associated with patches at the landscape level (Rempel, Kaukinen & Carr, 2012).

**Table 2:** Spatial correlation between variables (*N* = 31) in Scenario #1.

	1	2	3	4	5	6	7	8	9
1. *AREA* (ha)	–								
2. *DIST* (m)	−0.591***	–							
3. *ARD* (m)	−0.994**	0.582**	–						
4. *ED* (m/ha)	−0.375*	−0.039	0.425*	–					
5. *AWMSI* (–)	−0.187	−0.149	0.223	0.499**	–				
6. *MPFD* (–)	0.328	−0.073	−0.346	−0.278	−0.184	–			
7. *NumP* (#)	−0.486**	−0.024	0.519**	0.726***	0.307	−0.708***	–		
8. *MPS* (ha)	0.438*	−.052	−0.467**	−0.685***	−0.272	0.793***	−0.945***	–	
9. *VOL* (m^3^)	0.146	−0.062	−0.137	0.072	−0.072	−0.032	−0.023	−0.032	–
*M*	67.86	144.51	182.68	238.24	3.33	1.16	21.48	3.83	31,870.35
*SD*	3.69	23.60	26.26	21.08	0.20	0.02	5.18	0.83	22,542.68

**Notes:**

*AREA*, surface area below 40 dB(A) in noise models; *DIST*, minimum distance from the noise source to the 40 dB(A) contour line; *ARD*, average radius or radial distance of a circle of equal surface area than the sum of point receivers where SPL resulted higher than 40 dB(A); *ED*, edge density; *AWMSI*, area-weighted mean shape index; *MPFD*, mean patch fractal dimension; *NumP*, number of patches; *MPS*, mean patch size; *VOL*, the timber volume included in *AREA*; *M*, mean; *SD*, standard deviation.

* *p* < 0.05.

** *p* < 0.01.

*** *p* < 0.001.

### Predicting buffer protection distances

**Figure 4:**
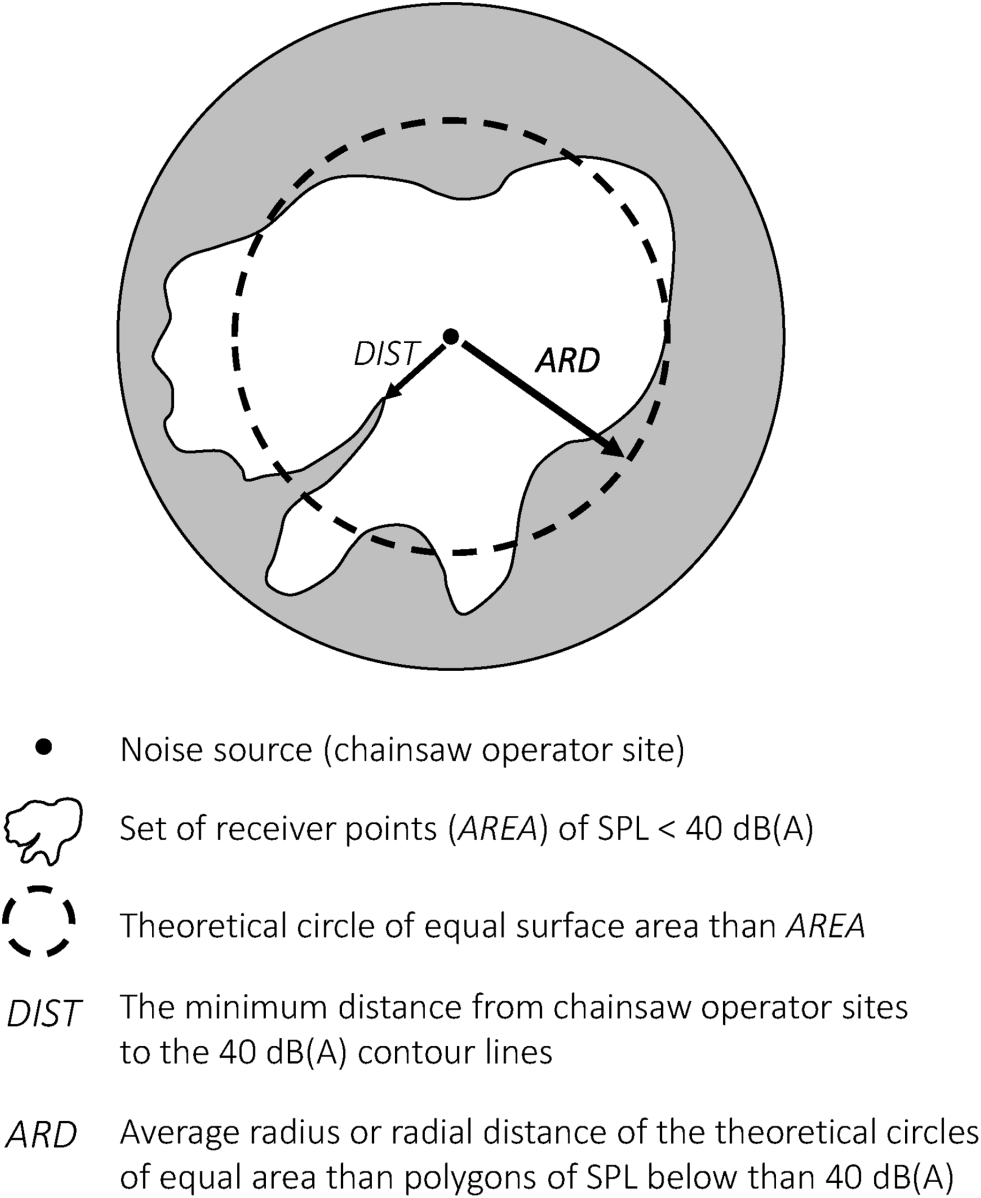
Graphical representation of the *ARD* index concept (based on sound pressure levels).

The reference value of 40 dB(A) was considered as being a noise level threshold that usually causes responses in wildlife (Shannon et al., 2016) and, particularly, the findings of Iglesias-Merchan, Diaz-Balteiro & De la Puente (2016) in relation to Cinereous vulture nesting in the nearby valley of Lozoya (Region of Madrid, Spain) were noted. Therefore, we calculated the surface area (*AREA*, in hectares) in which the predicted SPL resulted below 40 dB(A) within the 500 m buffer and the minimum distance (*DIST*, in meters) from the noise source to the 40 dB(A) contour line. In addition, we also calculated the average radius or radial distance (*ARD*) of a circle of equal surface area as the sum of point receivers, in which the SPL resulted higher than 40 dB(A) (Fig. 4). This represented the theoretical exclusion area for Cinereous vulture nesting because of its excessive exposure to SPL. We hypothesized that the results obtained could suggest a reduction in the current BPD criteria and a change in forest management plans in the case of allowing timber logging not only during the current 3 month period (fall) within CMA zones.

Lastly, we called *VOL* (m^3^) the timber volume included in *AREA* (ha) without exceeding a SPL of 40 dB(A) at distances shorter than 500 m, because this could potentially represent a spatial and/or temporary increase in timber productivity for forest planners, depending on the result of the average *DIST* or *ARD* values in stands. In the case of *DIST* values shorter than 100 m, this would mean the possibility (in terms of SPL) of felling new trees included in RMA zones, which implies a new spatial and temporal possibility. In the case of *DIST* values shorter than 500 m, that would only mean a new temporal possibility of timber logging throughout the year instead of the temporal cutting restrictions in CMA zones, which currently are only allowed in the fall.

Regarding the second objective of the study, a stepwise multiple linear regression was conducted to find a way to predict *DIST, ARD* or *AREA* values based on six variables (three forest and three orographic ones of the stands) while simultaneously removing those that were not important. In addition to the above mentioned orographic variables (*ELEV*, *ASPECT* and *SLOPE*), we considered three more variables for defining the forest tree cover: the surface area of the stands (*SURF*, in ha), basal area (*G*, in m^2^/ha) and density of trees (*N*, in number of trees/ha). Data were obtained from the current forest management plan. In this way it will be more feasible for forest planners to predict RMA and CMA zones around a nest site when they are designing a forest management plan. These analyses were performed with SPSS v. 24.0 (IBM, Armonk, NY, USA).

### Data analysis

We followed a clustering-based strategy to identify stands with equivalent noise propagation profiles that consisted of three steps (Fig. S1); data collection, stand clustering and test of means between cluster groups to evaluate dissimilarities.

The first step consisted of collecting the landscape analysis data of noise propagation throughout the stand surface. In the second step, the data obtained from the landscape analysis of noise maps were analyzed by means of multivariate analysis; the hierarchical cluster analysis (HCA), using the Ward’s linkage method, was employed to categorize stands in accordance with their noise propagation characteristics. Cluster analysis is a powerful tool which can effectively group similar objects while ensuring their distinction from other grouped objects (Li et al., 2018). In this study, the Euclidean distance was used as the dissimilarity measurement because it is the best known and most often used way of calculating the distance between samples (da Silva Torres, Garbelotti & Neto, 2006). In relation to determining the number of partitions, we adopted the minimum value of the indicator Akaike’s information criterion (AIC) from a specified range of between one and 15 clusters.

In order to identify the most relevant landscape variables, we conducted a preliminary Pearson correlation test to illustrate the strength of the relationship between them (Tables 2 and 3). This exploration revealed that the variables *AREA, ARD, ED, NumP, MPS* and *VOL* were correlated with *DIST, AWMSI* and *MPFD* in Scenario #1 (Table 2) and Scenario #2 (Table 3), so we decided to exclude *AREA, ARD, ED*, *NumP, MPS* and *VOL* from the HCA in both scenarios.

**Table 3:** Spatial correlation between variables (*N* = 31) in Scenario #2.

	1	2	3	4	5	6	7	8	9
1. *AREA* (ha)	–								
2. *DIST* (m)	−0.423*	–							
3. *ARD* (m)	−0.999***	0.419*	–						
4. *ED* (m/ha)	0.259	−0.253	−0.269	–					
5. *AWMSI* (–)	0.014	−0.187	−0.036	0.624***	–				
6. *MPFD* (–)	0.296	0.076	−0.308	0.003	0.031	–			
7. *NumP* (#)	−0.092	−0.260	0.599	0.651***	0.314	−0.634**	–		
8. *MPS* (ha)	0.019	0.332	−0.024	−0.611***	−0.281	0.727**	−0.927**	–	
9. *VOL* (m^3^)	0.382*	−0.212	−0.379*	0.183	−0.064	0.166	−0.035	−0.035	–
*M*	31.13	224.30	387.04	227.79	3.20	1.17	20.19	4.07	14,854.39
*SD*	8.50	51.21	34.32	22.76	0.18	0.02	4.73	0.88	11,830.90

**Notes:**

*AREA*, surface area below 40 dB(A) in noise models; *DIST*, minimum distance from the noise source to the 40 dB(A) contour line; *ARD*, average radius or radial distance of a circle of equal surface area than the sum of point receivers where SPL resulted higher than 40 dB(A); *ED*, edge density; *AWMSI*, area-weighted mean shape index; *MPFD*, mean patch fractal dimension; *NumP*, number of patches; *MPS*, mean patch size; *VOL*, the timber volume included in *AREA*; *M*, mean; *SD*, standard deviation.

* *p* < 0.05.

** *p* < 0.01.

*** *p* < 0.001.

In the third step, the selected variables and *VOL* mean values obtained from the cluster partitions were compared to assess the potentially significant influence of noise management in timber harvesting within each scenario. Once all the data did not meet the assumptions of normal distribution (Kolmogorov–Smirnov test) and the homogeneity of variance (Levene test), we used the nonparametric Mann–Whitney *U* test to compare the resulting cluster groups in Scenarios 1 and 2. All analyses were performed with SPSS v.24.0 (IBM, Armonk, NY, USA).

## Results

### Noise maps

**Figure 5:**
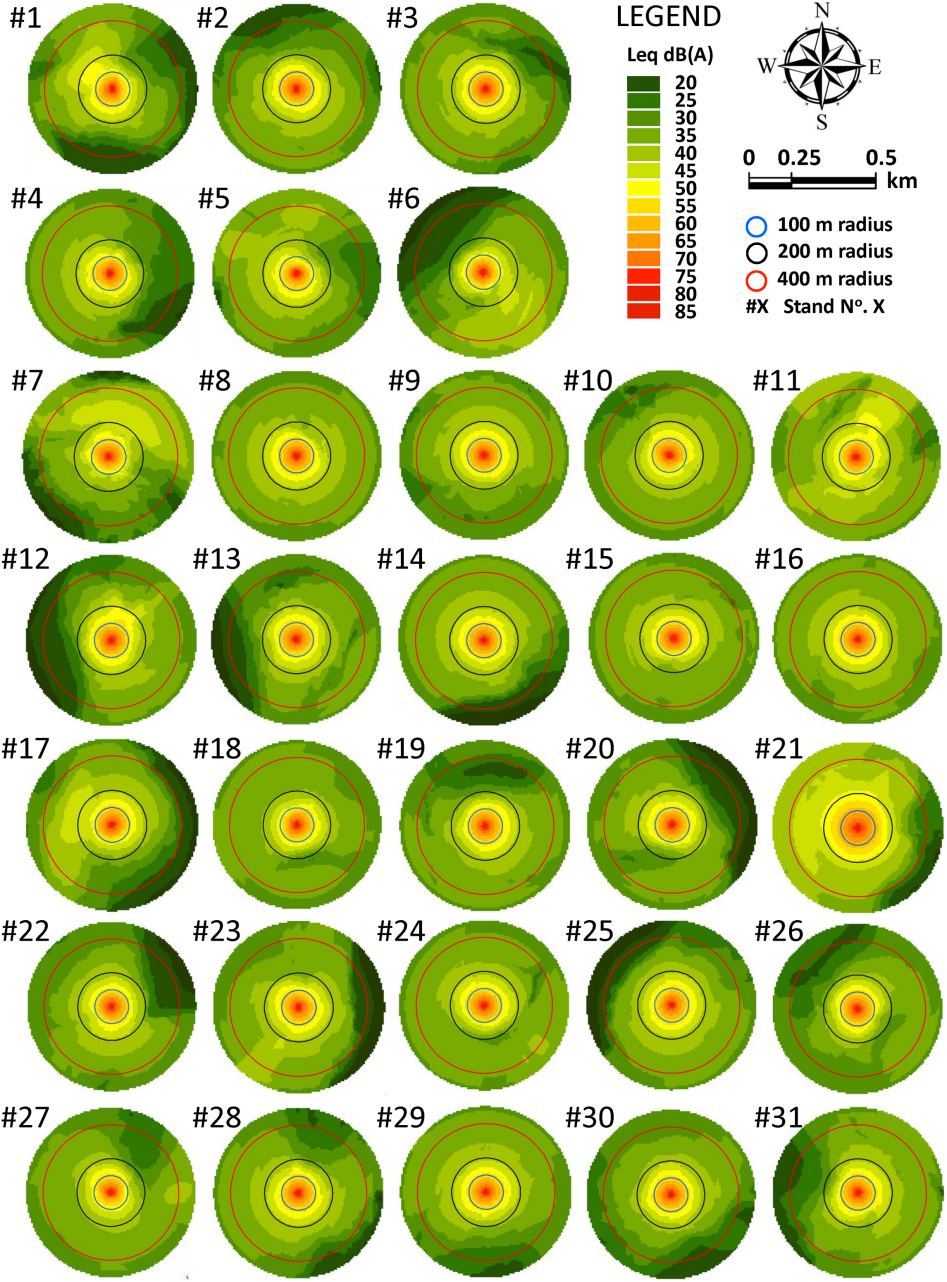
Noise maps for tree-felling located at the centroid point of the 31 stands in Scenario 1.

**Figure 6:**
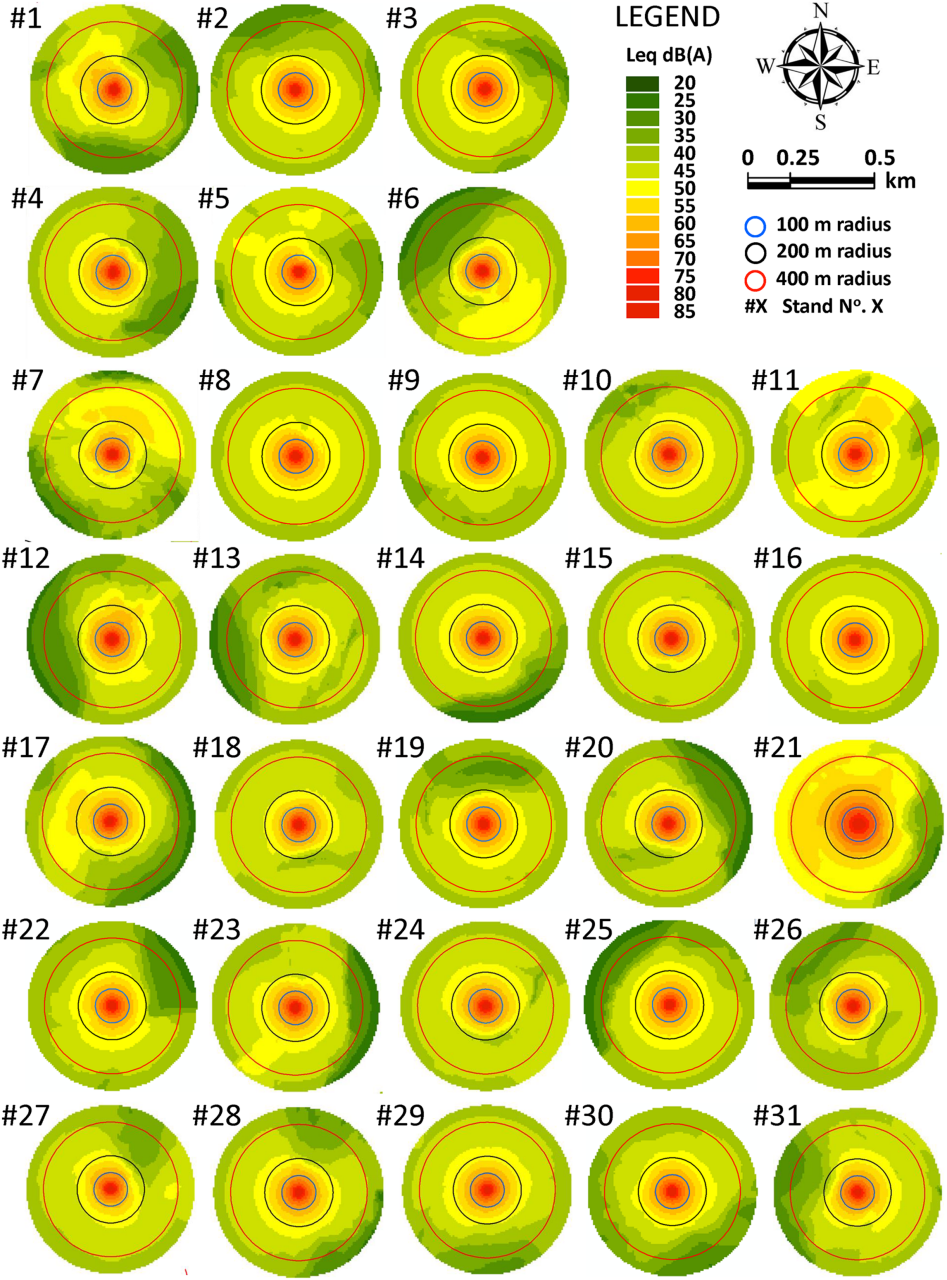
Noise maps for tree-felling located at the centroid point of the 31 stands in Scenario 2.

All the resulting 62 noise maps, which is the same as saying all the studied stands, included areas exposed to SPL of below 40 dB(A) within the 500 m circle around each stand centroid (Figs. 5 and 6), the location where we modeled the effect of a chainsaw operator felling a tree. In total, the sum of the surfaces of stands exposed to SPL of below 40 dB(A) was 2,103.53 ha (*M* = 67.86, *SD* = 3.69) in Scenario 1. This is approximately 86% of the area comprised within the 500 m modeled buffers and, taking into account the stocking rate (number of trees per hectare) in stands, this represents an average increase in timber volume of 31,870.35 m^3^ (*SD* = 22,542.68), which may potentially be unaffected by RMA or CMA restrictions.

In Scenario 2, the sum of the surfaces of stands exposed to SPL of below 40 dB(A) was 965.05 ha (*M* = 31.13, *SD* = 8.50) in. This is approximately 40% of the area comprised within the 500 m of modeled buffers. In this scenario, bearing in mind the number of trees per hectare in the stands, this percentage of area would mean an average increase in timber volume of 14,854.39 m^3^ (*SD* = 11,830.90), which could potentially be unaffected by RMA or CMA restrictions.

### The cluster analysis

#### Scenario 1

**Figure 7:**
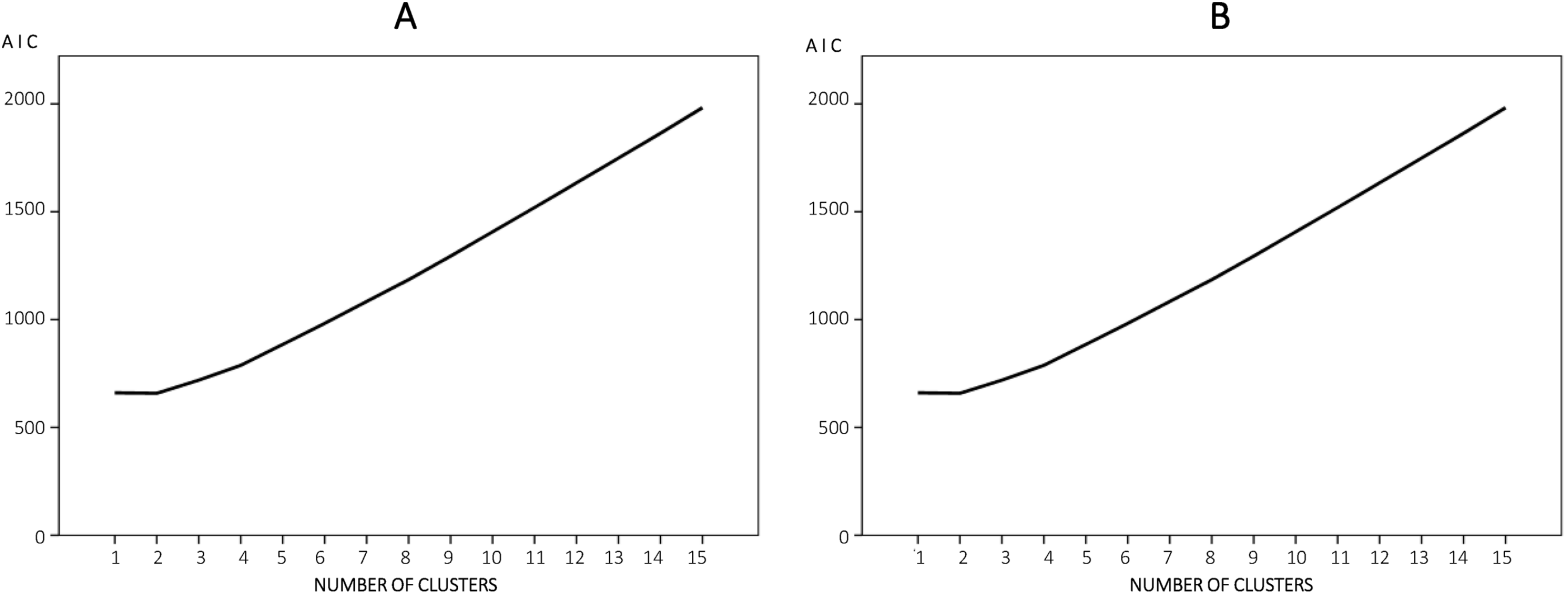
Akaike information criterion (AIC) and number of clusters. (A) Scenario 1. (B) Scenario 2.

A partition into two clusters statistically significantly different resulted in Scenario 1 (Fig. 7A). The first group was called cluster #1 (Table 4), and it was composed of 10 stands. The second group was called cluster #2 and it was composed of 21 stands. The statistically significant difference between both clusters (*U* (29) = 0.000, *p* < 0.001 (two-tailed)), was based on the variable *DIST*, since all the values in one subsample were strictly higher than all those in the other group. *DIST* median values from the chainsaw operator to the closest contour line of 40 dB(A) in cluster #1 (*M* = 119.15, *SD* = 7.40) were 30.38 m shorter than *DIST* median values in cluster #2 (*M* = 156.58, *SD* = 18.27). It is worth remembering that *ARD* was significantly correlated with the variable *DIST* and, now, *DIST* median values in cluster #1 (*M* = 174.41, *SD* = 18.93) and cluster #2 (*M* = 186.62, *SD* = 28.69) also showed a statistically significant difference (*U* (29) = 52.00, *p* = 0.025).

**Table 4:** Cluster membership and main results of spatial pattern of noise propagation through stands in Scenario 1.

*STAND* (ID)	*AREA* (ha)	*DIST* (m)	*ARD* (m)	*AWMSI* (no units)	*MPFD* (no units)	*VOL* (m^3^)	Cluster membership
#1	66.96	120.93	192.07	3.52	1.13	19,693.87	1
#2	68.67	156.54	177.34	3.10	1.14	24,050.00	2
#3	68.35	169.49	180.19	3.61	1.17	27,365.12	2
#4	69.67	120.03	168.12	3.21	1.16	25,842.02	1
#5	69.78	107.99	167.08	3.33	1.17	24,516.15	1
#6	69.94	107.33	165.55	3.16	1.16	24,115.78	1
#7	63.34	114.36	220.03	3.63	1.14	21,147.30	1
#8	68.55	126.32	178.41	3.28	1.19	10,417.08	1
#9	68.28	155.48	180.80	3.41	1.14	73,644.46	2
#10	68.56	150.14	178.32	3.30	1.16	11,237.67	2
#11	62.86	146.54	223.48	3.78	1.13	19,868.40	2
#12	67.67	150.86	186.10	3.32	1.19	101,033.91	2
#13	68.95	149.44	174.81	3.44	1.14	24,639.65	2
#14	68.21	165.91	181.42	3.10	1.19	9,651.72	2
#15	69.44	123.18	170.29	3.57	1.14	40,468.58	1
#16	67.68	170.08	186.01	3.32	1.16	22,856.59	2
#17	65.46	140.79	204.12	3.51	1.16	41,972.22	2
#18	70.35	146.23	161.56	3.19	1.16	38,833.78	2
#19	69.28	147.73	171.78	3.61	1.16	16,775.11	2
#20	69.75	130.40	167.37	3.52	1.16	64,061.78	1
#21	50.47	222.93	298.97	3.27	1.14	14,184.26	2
#22	69.15	147.17	172.98	3.16	1.16	6,289.16	2
#23	68.50	140.05	178.86	2.96	1.14	94,173.85	2
#24	69.34	146.35	171.22	3.37	1.17	38,413.26	2
#25	67.95	168.21	183.69	3.25	1.19	22,492.27	2
#26	70.51	121.95	159.98	3.48	1.20	26,729.49	1
#27	70.98	118.98	155.23	3.11	1.17	27,786.51	1
#28	68.64	152.70	177.61	3.27	1.15	29,470.83	2
#29	68.15	163.84	181.95	3.06	1.16	27,030.14	2
#30	68.87	162.83	175.53	3.19	1.14	32,181.80	2
#31	69.22	134.90	172.33	3.25	1.15	27,038.24	2

**Note:**

*AREA*, surface area below 40 dB(A) in noise models at distances shorter than 500 m from the chainsaw operator; *DIST*, minimum distance from the noise source to the 40 dB(A) contour line; *ARD*, average radius or radial distance of a circle of equal surface area than the sum of point receivers where SPL resulted higher than 40 dB(A); *AWMSI*, Area-weighted mean shape index; *MFD*, Mean patch fractal dimension; *VOL*, timber volume (m^3^) included in *AREA* (ha).

On the other hand, the partitions in Scenario 1 indicated that *AWMSI* median values in cluster #1 (*M* = 3.38, *SD* = 0.19) were almost equal to those included in cluster #2 (*M* = 3.31, *SD* = 0.20). In fact, there were no statistically significant differences in *AWMSI* (*U* (29) = 78.50, *p* = 0.268) values between cluster partitions and *MPFD* (*U* (29) = 89.50, *p* = 0.519). Also *MPFD* median values in cluster #1 (*M* = 1.16, *SD* = 0.02) were just the same as those included in cluster #2 (*M* = 1.16, *SD* = 0.02) and there were no statistically significant differences in *MPFD* (*U* (29) = 89.50, *p* = 0.519) values between both clusters. In short, both shape metrics *AWMSI* and *MPFD* values support the premise that patches’ shape is quite regular in both clusters.

Finally, as a consequence of considering the actual growing stock in each stand, *VOL* median values between both cluster partitions were assessed, but the Mann–Whitney *U* test showed a non-statistically significant difference between both cluster #1 (*M* = 28,477.86, *SD* = 14,576.56) and cluster #2 (*M* = 33,486.83, *SD* = 25,654.43), despite the significant differences in *DIST* values and its significant correlation with the variable *AREA*.

#### Scenario 2

Two clusters were also determined following the AIC in Scenario 2 (Fig. 7B). The first group was called cluster #1 (Table 5) and it was composed of 14 stands, whose characteristics statistically differed from others, included in a second group called cluster #2 (composed of 17 stands). The statistically significant difference between both clusters (*U* (29) = 0.000, *p* < 0.001 (two-tailed)), was based on the variable *DIST*, since all the values in one group were higher than all those in the other group, the same as in Scenario 1. *DIST* median values from the centroid of the stand to the closest contour line of 40 dB(A) in cluster #1 (*M* = 191.18, *SD* = 16.42) were 68.25 m shorter than *DIST* median values in cluster #2 (*M* = 276.74, *SD* = 42.62). Also, like in Scenario 1, *ARD* median values in cluster #1 (*M* = 374.16, *SD* = 28.73) and cluster #2 (*M* = 407.43, *SD* = 33.49) also showed a statistically significant difference (*U* (29) = 51.00, *p* = 0.010).

**Table 5:** Cluster membership and main results of spatial pattern of noise propagation through stands in Scenario 2.

*STAND* (ID)	*AREA* (ha)	*DIST* (m)	*ARD* (m)	*AWMSI* (no units)	*MPFD* (no units)	*VOL* (m^3^)	Cluster membership
#1	38.78	179.87	355.80	3.53	1.18	11,405.74	1
#2	36.78	226.42	364.63	3.02	1.16	12,881.30	1
#3	35.14	202.65	371.72	3.38	1.16	14,068.91	1
#4	39.59	184.13	352.16	3.08	1.16	14,684.73	1
#5	24.77	162.34	413.75	3.05	1.13	8,702.57	1
#6	34.87	166.45	372.88	3.02	1.15	12,023.41	1
#7	27.33	191.66	403.78	3.47	1.14	9,124.65	1
#8	20.07	402.46	431.45	3.04	1.20	3,049.90	2
#9	30.83	261.37	389.74	3.17	1.14	33,252.18	2
#10	27.17	249.29	404.41	3.22	1.16	4,453.44	2
#11	12.23	257.07	459.46	3.57	1.14	3,865.58	2
#12	38.50	194.43	357.05	3.20	1.19	57,481.98	1
#13	42.28	216.50	339.78	3.36	1.15	15,108.98	1
#14	30.19	259.03	392.34	3.00	1.20	4,271.89	2
#15	23.49	251.16	418.64	3.19	1.15	13,689.62	2
#16	21.91	294.84	424.61	3.05	1.13	7,399.35	2
#17	35.10	209.72	371.89	3.41	1.17	22,505.73	1
#18	24.57	184.27	414.52	3.17	1.17	13,562.84	1
#19	36.38	197.59	366.38	3.49	1.17	8,808.87	1
#20	39.72	179.21	351.57	3.29	1.18	36,480.77	1
#21	12.73	285.58	457.72	3.39	1.17	3,577.68	2
#22	38.64	204.32	356.42	3.04	1.16	3,514.29	1
#23	27.16	251.71	404.45	2.90	1.16	37,339.59	2
#24	17.84	189.65	439.60	3.13	1.16	9,883.08	1
#25	35.41	289.93	370.57	3.15	1.16	11,721.14	2
#26	45.62	191.91	323.76	3.35	1.21	17,293.99	1
#27	28.81	176.92	397.90	3.01	1.18	11,278.24	1
#28	35.23	197.66	371.34	3.12	1.17	15,126.13	1
#29	30.54	260.79	390.92	2.97	1.15	12,112.99	2
#30	41.18	257.60	344.89	3.11	1.16	19,242.72	2
#31	32.19	176.77	384.15	3.20	1.15	12,573.84	1

**Note:**

*AREA*, surface area below 40 dB(A) in noise models at distances shorter than 500 m from the chainsaw operator; *DIST*, minimum distance from the noise source to the 40 dB(A) contour line; *ARD*, average radius or radial distance of a circle of equal surface area than the sum of point receivers where SPL resulted higher than 40 dB(A); *AWMSI*, Area-weighted mean shape index; *MFD*, Mean patch fractal dimension; *VOL*, timber volume (m^3^) included in *AREA* (ha).

The partitions in Scenario 2 indicated that *AWMSI* median values in cluster #1 (*M* = 3.23, *SD* = 0.18) were almost equal to those included in cluster #2 (*M* = 3.15, *SD* = 0.19), and there were no statistically significant differences in *AWMSI* (*U* (29) = 84.50, *p* = 0.236) values between cluster partitions. Also, *MPFD* median values in cluster #1 (*M* = 1.17, *SD* = 0.02) were almost the same as those included in cluster #2 (*M* = 1.16, *SD* = 0.02), and *MPFD* (*U* (29) = 88.50, *p* = 0.306) values between cluster partitions, like in Scenario 1. This means that the spatial pattern of sound propagation throughout the forest is very similar in terms of the shape of the patches and the metrics used to describe the resulting SPL polygons in noise maps. In short, both shape metrics *AWMSI* and *MPFD* values reflect that the shape of patches is quite regular in both clusters. Although *AWMSI* values may lead some to say that the patches are slightly less irregular in Scenario 2 than in Scenario 1, but it would be a non-significant difference. Therefore, the significance of spatial pattern results in determining the nature of SPL due to tree logging seems to be related to the size of patches rather than shape and edge metrics when comparing data from Scenarios 1 and 2. In relation to the size of patches, it is worth noting that *AREA* mean values in Scenario 1 are higher than double their size in Scenario 2. This is, obviously, correlated with the sound power level of the noise source in each scenario (the lower the sound power level, the higher the *AREA* polygons size).

Finally, as a consequence of considering the actual timber stock in each stand, *VOL* median values between both cluster partitions were assessed, but the Mann–Whitney *U* test showed a non-statistically significant difference between both cluster #1 (*M* = 16,132.11, *SD* = 12,071.70) and cluster #2 (*M* = 12,831.24, *SD* = 11,661.54), despite the significant differences in *DIST* values and its significant correlation with the variable *AREA*.

### Predicted buffer distances

#### Scenario 1

The stepwise multiple linear regression found that no variables were entered into the equation in Scenario 1. That means that *DIST* attenuation of SPL made by chainsaws at felling sites cannot be predicted as a function of forest and orographic variables, and the explanation could rather lie in the inverse square law of sound propagation. In other words, given a chainsaw with a known sound power level and the spectral content of the tree-felling signal, forest planners could estimate the suitable forest area that would potentially be affected, or not, by logging restrictions (CMA) in mountain terrains as easily as it could be estimated in flat terrains.

#### Scenario 2

On the other hand, the stepwise multiple linear regression found a significant regression equation (*F* (2, 28) = 5.726 *p* = 0.008), with an *R*^2^ of 0.240 in Scenario 2 (Table 6). In particular, *DIST* can be predicted as a function of the terrain slope (*SLOPE*) and the stand’s tree density (*N*), where the ground slope was measured as a percentage and tree density in number of trees per hectare:

(1) $$DIST\, = \,256.91\, + \,\left( {0.153 \times \left( N \right)} \right)\, - \,\left( {2.659\, \times\,\left( {SLOPE} \right)} \right)$$

**Table 6:** Summary of stepwise regression analysis for variables predicting *DIST* values (*N* = 31) in Scenario 2.

Variable	*B*	*SE B*	β	*t*	*p*
*SLOPE*	−1.854	0.770	−0.408	−2.409	0.023
*N*	0.153	0.069	0.394	2.208	0.036

**Note:**

*SLOPE*, slope of the ground (in percentage); *N*, the stand’s tree density (in number of trees/ha); *B*, the unstandardized beta; *SE B*, the standard error for the unstandardized beta; β, the standardized beta; *t*, the *t*-test statistic; *p*, the probability value.

That means that forest planners could estimate the minimum distance on maps from a tree-felling site to the LApeak level of 40 dB(A) contour line (Eq. (1)) due to the peak noise caused by tree-felling (Scenario 2), unlike the first case (Scenario 1) within the study area.

Nevertheless, these results were not the same when we tried to predict the theoretical variable *ARD* (there was no discernible linear relationship between *ARD* and any of the predictors), which may highlight noise modeling as being needed to accurately meet the challenge of estimating the radius of the theoretical circle that equals the value of *AREA* for each case study.

## Discussion

On the one hand, it is worth mentioning that *DIST* and *ARD* indices always resulted in being longer than 100 m. It was found that SPL exceeded 40 dB(A) beyond 100 m in all the 62 noise models in both scenarios. Nevertheless, in viewing the noise maps of the stands, it is easy to perceive spatial differences between stands in both Scenario 1 and Scenario 2. This finding is not a novelty in mountainous terrain, in which SPLs do not propagate as parallel contour lines because of the obstacles created by the terrain itself (Iglesias-Merchan & Diaz-Balteiro, 2013). However, as a result, a statistically significant difference was found in spatial patterning of noise (i.e., the variable *DIST*) between groups of stands in both Scenario 1 and Scenario 2. Mean *DIST* values ranged between 119.15 m in cluster #1 of Scenario 1 and 276.74 m in cluster #2 of Scenario 2. Therefore, our results agree with the field work of Potočnik & Poje (2010), that, empirically, found that the total chainsaw power equals the wind noise at distances of 140 m, and at a distance of approximately 250 m, it matches the forest silence background noise. The importance of this finding is that all the stands could tolerate a reduction in logging restrictions in terms of SPL, because chainsaw noise is significantly attenuated at approximately 120–275 m from the tree-felling site within the study area. But one question would still remain: how can we reduce spatial restrictions?

On the other hand, given a sound power level of chainsaws, their operators could work without exceeding the mentioned SPL threshold of 40 dB(A) at shorter distances than 500 m throughout all the seasons. This finding would permit a significant increase in the total allowable annual cut surface. In this sense, the variable *AREA* would offer an answer to the previous question. According to results in Scenario 1, logging could be potentially planned in 86.4% of the surfaces that could currently be considered as CMA zones when applying a distance (in meters) criterion instead of a SPL (in decibels) one. This currently causes the restriction of forest works to a short period of only 3 months (the fall) every year in approximately 56% of the Scots pine forest surface (4,211 ha) in our study area. Considering Scenario 2, this trend should be more nuanced and logging could be potentially planned in no more than 39.6% of the surfaces that could currently be considered as CMA zones. The quantitative difference between both scenarios could be controversial because of the novelty of its focus.

In this sense, it is important to note that there is often confusion between the term *L*_max_ (maximum sound level, usually expressed in dB(A)) and the term peak (in dB usually with *C* frequency weighting), despite their being very different. *L*_max_ is the highest sound level over a measuring period (in slow response) meanwhile the peak SPL is the actual peak level of the pressure wave (Cirrus, 2015). The latter is the metric that we have used. Indeed, peak level is a metric of interest for brief noise events as it measures peak instantaneous pressure; in particular, in the case of impulse noise or single bursts of a short duration that may cause peak levels that are 15 dB higher than background noise (Starck, Toppila & Pyykko, 2003), although this requires relatively sophisticated instrumentation (Pater, Grubb & Delaney, 2009). All this deserves to be mentioned in order to prevent any misunderstandings, because some methodological protocols have been questioned in relation to studies aimed at assessing the potential effects of anthropogenic noise on wildlife (Dutilleux, 2012). This might explain the lack of evidence in the literature on the potential effects of impulsive brief events separately from the complete fluctuating noise signal in which they are embedded.

In many countries, one of the strategies developed to retain a large proportion of the mature wildlife community is to define areas where final cuttings are not allowed (Holmes et al., 2012; Ezquerro, Pardos & Diaz-Balteiro, 2019). So, our findings could be regarded as being attractive because of their potential socio-economic consequences, without forgetting that biodiversity conservation is an increasingly required objective in timber production (Ezquerro, Pardos & Diaz-Balteiro, 2016). Although combining the retained areas with more intensive management can offer results that compete economically with traditional management forms (Bravo & Diaz-Balteiro, 2004), it should also be noted no statistically significant difference in timber productivity increase (*VOL*) between stands was found in the case of considering a theoretical reduction in spatial logging restrictions. These details are relevant when the socio-economic vulnerability of rural mountainous areas due to external pressures and constraints could have induced land abandonment, which frequently causes unpredicted and spatially diverse environmental impacts (MacDonald et al., 2000).

However, habitat alteration and human disturbance are widely regarded as major factors contributing to the decline of forest animals (Schmiegelow & Mönkkönen, 2002; Poirazidis et al., 2004). Studies that have isolated noise from potentially confounding variables have evidenced that noise alone can negatively affect wildlife (e.g., behavior alteration, habitat quality reduction, physiological effects) (Shannon et al., 2016). Nevertheless, a gap exists in the literature to relate the partial distribution of the acoustic energy from anthropogenic sources and its potential effects on wildlife in terrestrial ecosystems. In addition, the outcome of habitat degradation requires the identification of habitat types and species of concern (Schmiegelow & Mönkkönen, 2002). For instance, the distance which an animal flees from the disturbing source may depends on a variety of factors (i.e., type of disturbance, habitat, season, habituation, visibility, etc.) (Theobald, Miller & Hobbs, 1997; González et al., 2006). In this sense, Legagneux & Ducatez (2013) reported that common European birds changed their flight initiation distances in response to vehicles according to the vehicle’s speed (which directly determines the sound power level of the infrastructure). Moreover, chainsaws have been found to be more disturbing to Spotted owls than helicopter flights at comparable distances (Potočnik & Poje, 2010).

In forest science, logging disturbances to wildlife are commonly considered as a “hotchpotch,” although researchers have been unable to distinguish between several causes of disturbance due to logging until now (Plumptre & Johns, 2001). Nevertheless, the strategy of retained areas seems to be justified from the point of view of conservation. Even more once the number of reproductive couples of Cinereous vulture is known within the study area (Ezquerro, Pardos & Diaz-Balteiro, 2019). However, some doubt arise on their monetary cost in terms of loss of timber production of the forest, an area in which our findings could increase this perception, although this doubt could change significantly if we take into consideration the results of the *ARD* indicator. The *ARD* index represents the same, easy spatial concept as the buffer zones, but it is based on a calculated SPL and the particularities of sound propagation within a study area. In Scenario 1, mean *ARD* values ranged between 174.4 and 186.6 m in cluster #1 and cluster #2, respectively. These values are significantly higher than the current radius size of RMA zones (100 m). On the contrary, in Scenario 2, mean *ARD* values ranged between 374.16 and 407.43 m in cluster #1 and cluster #2 respectively. The significant increase in mean *ARD* values is due to the difference in the frequency spectrum of the sound event as well as the SPL of the noise source with respect to Scenario 1. In Scenario 2, *ARD* values are approximately 100 m shorter than the current radius size of CMA zones (500 m). Thus, comparing the size of traditional RMA and our results in Scenarios 1 and 2, it is possible to conjecture that an excess of size in CMA zones may be currently compensating for the scant size of RMA zones if it is intended to prevent disturbance due to forest logging.

Therefore, taking into account *ARD* values, it can be asserted that current practices are well oriented when forest managers decide to retain patches and buffer zones of several hundred meters in radius in order to prevent disturbance caused by forest logging operations, despite the scarcity of evidence and of specific indicators and threshold ranges in habitat alteration to verify the several causes of potential disturbance due to logging (Plumptre & Johns, 2001; Guénette & Villard, 2005). Nevertheless, on the basis of our results, it could be recommended to increase the radius of RMA zones for up to 200 m in order to prevent potential disturbance of nearby Cinereous vulture nest sites when using the chainsaw to cut down trees. This is especially in case the areas exposed to SPL below 40 dB(A) are excluded from CMA zones after consideration of the same findings of our work, that means that the radius of CMA zones could be reduced from 500 to 400 m. This proposal would allow a more flexible management of timber harvesting in a 100 m wide outer ring within current CMA zones. This outer ring represents 36% of the area of CMA zones, which includes a total approximate amount of 135,000 m^3^ of timber (*M* = 4,346.10, *SD* = 2,323.24). Obviously, behavioral monitoring of vultures during forest logging operations could help to determine the actual influence of the fluctuating noise of chainsaws against the impulsive noise when the trunk finally breaks and hits the ground. Thus, further research would be needed in this respect because there is a complete lack of published data regarding this issue.

Environmental noise pollution due to a variety of industrial noise sources has been thoroughly investigated on large scales in the literature (Guarnaccia, Mastorakis & Quartieri, 2011), and the combination of landscape perspective and noise mapping tools have been pointed to as providing a relevant working procedure to be considered by planners in areas where SPL should be managed (Iglesias-Merchan & Diaz-Balteiro, 2013; Aletta & Kang, 2015). But it is very striking that, until now, environmental noise pollution has not yet been quantified in sustainable forest management planning, despite the existence of some well-defined management plans that traditionally consider some kind of stocking to provide a suitable habitat for a number of listed species (Fiedler & Cully, 1995). However, in our work, we do not enter into the criteria of habitat selection for sensitive species, which may prefer, for instance, a minimum percentage of canopy cover (Hanski, Fenske & Niemi, 1996; North et al., 2017). Nevertheless, and taking into account the findings, this may be a controversial issue when the crucial argument for establishing restricted areas for timber logging is the potential capacity of human activities to disturb wildlife. The latter may require a more detailed assessment at population level, because forest animals may respond in a wide variety of ways (Rempel et al., 1997; Plumptre & Johns, 2001). Anyway, the effects of habitat alterations are scant because their study requires years or decades of environment monitoring. As a result, forest managers rarely adequately address the long-term capacity of long-living species to cope with environmental disturbance (Donázar et al., 2002).

Considering the methodology presented, noise modeling can be considered to be of assistance in sustainable forest planning. In addition, another advantage of the *ARD* index is that it can also be managed in the same way as current buffer-protected areas, by both forest managers and field operators on the ground. It should not be disregarded that large birds of prey frequently use a variable number of nests, but that their location is also known and inventoried in natural protected areas. Thus, it seems feasible for forest managers to handle a limited number of noise maps, like any other thematic cartography that they use, in order to conduct machinery movements through the forest. Therefore, particular forestry activities (e.g., harvesting) could be defined later on in detail (at the stand and operational level) and conveyed to chainsaw operators on a daily basis.

Our results cannot be directly extrapolated to every combination of forest machinery, species and study areas, because it would be necessary to model their particular SPL conditions, working-cycle duration, thresholds of response to disturbance and local orography conditions, among other factors. Further research may also be needed to address noise pollution in tactical forest management modeling in order to enable foresters to use noise indices in multi-criteria models (Ezquerro, Pardos & Diaz-Balteiro, 2019); thereby lies a challenge to develop new methods for planning environment-friendly and sustainable forest cuttings within participating landscapes (Turner, Fischer & Tzanopoulos, 2018).

## Conclusions

Our research reveals the potential use of noise mapping to model and predict BPD in order to manage noise disturbance due to forest operations at forest planning level. Our starting hypothesis was not confirmed because it was found that the spatial propagation of noise made by chainsaws at felling sites did not differ between stands even on a mountainous terrain. Indeed, results showed the statistical viability of proposing a categorization of the stands based on spatial patterns of noise propagation.

We developed a new indicator, the *ARD*, which represents the radius of the theoretical circle of equal surface area as the sum of point receivers around the noise source, where SPL resulted higher than a known threshold for the studied species (i.e., 40 dB(A) in the case of the Cinereous vulture nesting habitat).

Finally, two suggestions could be made with respect to the current restrictions to forest logging within the study area: (i) On the one hand, it is suggested to extend the current size of RMA zones from a 100 m radius to a 200 m one; (ii) on the other, temporary logging restrictions could be reduced from a 500 m radius to a 400 m one, in application of SPL (in decibels) criteria instead of the traditional BPD.

## Supplemental Information

10.7717/peerj.6922/supp-1 Supplemental Information 1 Outline of the three-step proposed strategy.

10.7717/peerj.6922/supp-2 Supplemental Information 2 Spatial statistics indices used in this study. Source: McGarigal & Marks, 1995.

10.7717/peerj.6922/supp-3 Supplemental Information 3 Raw data. Centroid (Group 0) and stands (Group 1) orographical data.

10.7717/peerj.6922/supp-4 Supplemental Information 4 Raw Data Scenario 1. Landscape, orographical and forest data at each stand’s centroid point in Scenario 1.

10.7717/peerj.6922/supp-5 Supplemental Information 5 Raw Data Scenario 2. Landscape, orographical and forest data at each stand’s centroid point in Scenario 2.

## Additional Information and Declarations

### Field Study Permissions

The following information was supplied relating to field study approvals (i.e., approving body and any reference numbers): Acoustic measurements in the Forest of Valsaín were made under a Certificate of Authorization of the Service of the Environment (Regional Government Delegation in Segovia, The Government of Castilla y León) awarded to ETSI Montes, Forestal y del Medio Natural, Universidad Politécnica de Madrid (Expte: PNSNG_SG_2017_0180).

### Data Availability

The following information was supplied regarding data availability: The raw data are available in the Supplemental Files.
