# Supplementary figures and images for: Incorporating acoustic objectives into Forest Management Planning when sensitive bird species are relevant
Source: PeerJ. 2019 May 16;7:e6922. doi: 10.7717/peerj.6922 (PMC6526013; doi:10.7717/peerj.6922)

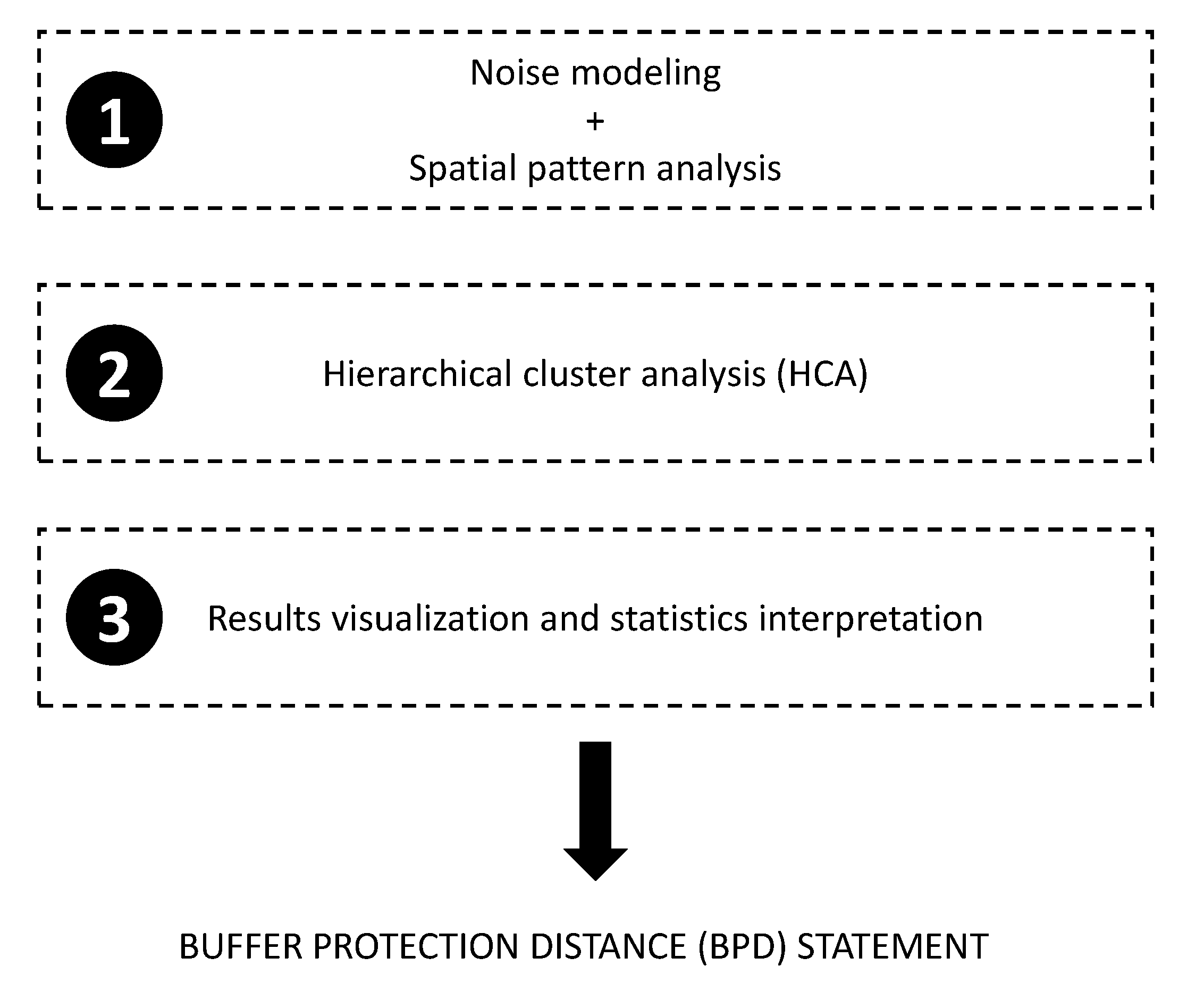

Supplement: Supplemental Information 1 [file peerj-07-6922-s001.png]
