# Supplementary material for: Incorporating acoustic objectives into Forest Management Planning when sensitive bird species are relevant
Source: PeerJ. 2019 May 16;7:e6922. doi: 10.7717/peerj.6922 (PMC6526013; doi:10.7717/peerj.6922)
Supplement: Supplemental Information 2 — Source: McGarigal & Marks, 1995. [file peerj-07-6922-s002.docx]

| Table S1.  Spatial statistics indices used in this study. | | | | |
| --- | --- | --- | --- | --- |
| **Metrics** | **Measure** | **Selected indices** | **Meaning** | **Equations** |
| Patch Density and Size Metrics | Landscape fragmentation and configuration | Number of patches, *NumP* (#) | Number of patches ($p_{t}$) at landscape level or for each individual class (if analyzed by class). Each class is represented by SPL categories defined by ranges of 5 dB(A). | $NumP=\sum_{t=1}^{n} p_{t}$ |
|  |  | Mean patch size, *MPS* (ha) | Average patch size ($a_{ij}$) analyzed by class or landscape level ($n_{i}$refers *NumP*). In this case *MPS* is analyzed at landscape scale. | $MPS=\frac{\sum_{j=1}^{n} a_{ij}}{n_{i}}$ |
| Edge Metrics | Amount, length, and distribution of edges between specific patch types | Edge density, *ED* (m/ha) | Patch perimeter or edge length (E) referred to the landscape total area (A) or each class area (*CA*). *CA* is the sum of areas of all patches ($a_{ij}$) belonging to a given SPL category of 5 dB(A) increments. | $ED=\frac{\sum_{i=1}^{n} E_{i}}{A or CA}$ |
| Shape Metrics | Geometric complexity | Area-weighted mean shape index, *AWMSI* (no units) | It is equal to 1 when all patches are circular (for vector files) or square (for raster files) and increases (without limit) when increasing patch shape irregularity ($p_{ij}$is the perimeter of patch *ij*) | $AWMSI=\sum_{j=1}^{n} \left[ \frac{p_{ij}}{minp_{ij}}\left( \frac{a_{ij}}{\sum_{j=1}^{n} a_{ij}} \right) \right]$ |
|  |  | Mean patch fractal dimension, *MPFD* (no units) | It approaches 1 for shapes with very simple perimeters (circles or squares) and approaches 2 for highly convoluted perimeters | $MPFD=\frac{\sum_{j=1}^{n} (\frac{{2ln p}_{ij}}{{ln a}_{ij}})}{n_{i}}$ |
| Source: McGarigal & Marks, 1995 | | | | |
